# Tizoxanide pyridine monosolvate

**DOI:** 10.1107/S1600536812016133

**Published:** 2012-04-21

**Authors:** Huaqin Zheng, Hui Deng, Yunyun Chen, Ding Li

**Affiliations:** a School of Pharmaceutical Science, Sun Yat-sen University, Guangzhou, 510006, People’s Republic of China

## Abstract

In the title compound [systematic name: 2-hy­droxy-*N*-(5-nitro-1,3-thia­zol-2-yl)benzamide pyridine monosolvate], C_10_H_7_N_3_O_4_S·C_5_H_5_N, the dihedral angle between the pyridine and benzamide rings is 80.55 (7)°. An intamolecular O—H⋯N hydrogen bond occurs in the tizoxanide. In the crystal, the components are linked by an O–H⋯N hydrogen bond, forming a zigzag chain along the *c* axis. Aromatic π–π inter­actions between inversion-related pyridine rings [centroid–centroid distance = 3.803 (6) Å] are also observed.

## Related literature

For the biological activity of tizoxanide, see: Rao *et al.* (2009 ▶); Gargala *et al.* (2000 ▶); Dubreuil *et al.* (1996 ▶); Ashton *et al.* (2010 ▶); Korba, Elazar *et al.* (2008 ▶); Zhao *et al.* (2010 ▶). For related structures and background to the bioactivity of tizoxanide, see: Pankuch & Appelbaum (2006 ▶); Stettler *et al.* (2003 ▶); Broekhuysen *et al.* (2000 ▶). For details on experimental methods used to obtain this form and analogues, see: Navarrete-Vazquez *et al.* (2011 ▶). For a pyridine-solvated forms, see: Dong *et al.* (2011 ▶). For additional literature on related tizoxanide thiazolide compounds, see: Megraud *et al.* (1998 ▶); Chan-Bacab *et al.* (2009 ▶); Korba, Montero *et al.* (2008 ▶); Stachulski *et al.* (2011*a* ▶ *b* ▶). For the biological activity of the anti-parasitic agent nitazoxanide {systematic name: [2-[(5-nitro-1,3-thiazol-2-yl)carbamoyl]phenyl]ethanoate}, see: Hemp­hill *et al.* (2006 ▶); Rossignol *et al.* (2006 ▶). For the structure of nitazoxanide, see: Bruno *et al.* (2010 ▶). For the effect of crystallization from different solvents on drug properties, see: Trask *et al.* (2004 ▶). 
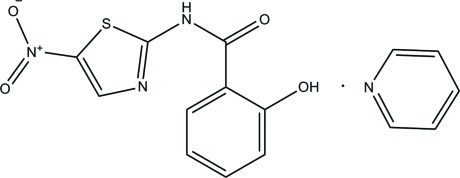


## Experimental

### 

#### Crystal data

C_10_H_7_N_3_O_4_S·C_5_H_5_N
*M* *_r_* = 344.35
Triclinic, 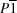

*a* = 6.9826 (3) Å
*b* = 10.0462 (5) Å
*c* = 11.8387 (7) Å
α = 102.998 (5)°
β = 99.037 (5)°
γ = 104.367 (4)°
*V* = 763.69 (7) Å^3^
*Z* = 2
Cu *K*α radiation
μ = 2.16 mm^−1^
*T* = 293 K
0.20 × 0.15 × 0.10 mm

#### Data collection

Agilent Xcalibur Onyx Nova diffractometer
Absorption correction: multi-scan (*CrysAlis PRO*; Agilent, 2011 ▶) *T*_min_ = 0.712, *T*_max_ = 0.806
4493 measured reflections
2481 independent reflections
2349 reflections with *I* > 2σ(*I*)
*R*_int_ = 0.016

#### Refinement

*R*[*F* ^2^ > 2σ(*F* ^2^)] = 0.028
*wR*(*F* ^2^) = 0.075
*S* = 1.08
2481 reflections
265 parameters
All H-atom parameters refined
Δρ_max_ = 0.20 e Å^−3^
Δρ_min_ = −0.25 e Å^−3^

### 

Data collection: *CrysAlis PRO* (Agilent, 2011 ▶); cell refinement: *CrysAlis PRO*; data reduction: *CrysAlis PRO*; program(s) used to solve structure: *SHELXS97* (Sheldrick, 2008 ▶); program(s) used to refine structure: *SHELXL97* (Sheldrick, 2008 ▶); molecular graphics: *OLEX2* (Dolomanov *et al.*, 2009 ▶); software used to prepare material for publication: *OLEX2*.

## Supplementary Material

Crystal structure: contains datablock(s) I, global. DOI: 10.1107/S1600536812016133/qm2062sup1.cif

Supplementary material file. DOI: 10.1107/S1600536812016133/qm2062Isup2.mol

Structure factors: contains datablock(s) I. DOI: 10.1107/S1600536812016133/qm2062Isup3.hkl

Supplementary material file. DOI: 10.1107/S1600536812016133/qm2062Isup4.cml

Additional supplementary materials:  crystallographic information; 3D view; checkCIF report

## Figures and Tables

**Table 1:** Hydrogen-bond geometry (Å, °)

*D*—H⋯*A*	*D*—H	H⋯*A*	*D*⋯*A*	*D*—H⋯*A*
N7—H7⋯O1	0.85 (2)	1.95 (2)	2.6248 (16)	135.9 (18)
O1—H25⋯N8	0.94 (2)	1.64 (2)	2.5671 (16)	175 (3)

**Fig. 1.**
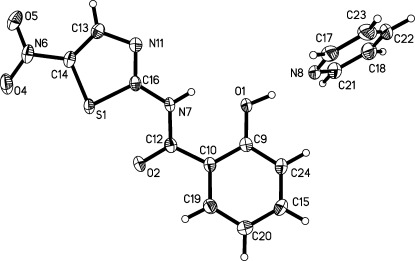
A view of the molecular structure of the title compound (I). The displacement ellipsoids are at the 50% probability level.

**Fig. 2.**
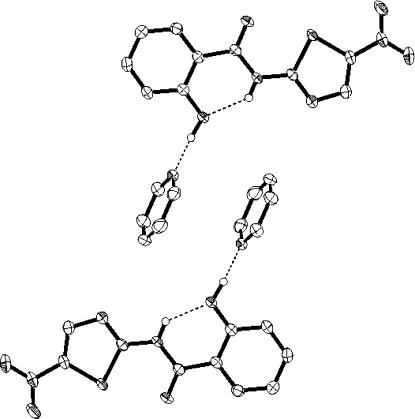
The two-dimensional plane formed by the hydrogen bonds of the molecules; dashed lines represent hydrogen bonds and some of the H atoms have been omitted for reasons of clarity.

**Fig. 3.**
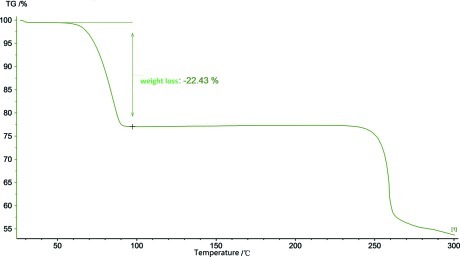
The thermogravimetric analysis of the title compound showing a one-step molecular weight loss of 22.43% corresponding to one pyridine molecule.

## supplementary crystallographic information

### Comment

Nitazoxanide was first developed as an anti-parasitic agent, and marketed in the USA since 2002 (Stachulski *et al.*, 2011*a*). In humans, once orally administered, nitazoxanide is hydrolyzed in plasma to its active metabolite tizoxanide (TIZ), which is 99% protein bound (Broekhuysen *et al.*, 2000; Ashton *et al.*, 2010). Nitazoxanide exhibits a broad spectrum of activities against intracellular and extracellular protozoa, helminthes, aerobic and anaerobic bacteria, and viruses infecting humans and animals (Hemphill *et al.*, 2006; Rossignol *et al.*, 2006; Korba, Montero *et al.*, 2008; Zhao *et al.*, 2010; Stachulski *et al.*, 2011*b*).

In the present study, tizoxanide was prepared *via* deacetylation of nitazoxanide in pyridine and it crystallized as a 1:1 ratio complex with pyridine as crystals. Thermogravimetric analysis was performed to study the thermal stability of the title complex, which indicated a one-step molecular weight loss of 22.43% corresponding to one pyridine molecule in the temperature range of 333–373 K, confirming a 1:1 ratio complex of tizoxanide-pyridine (theoretical weight loss 22.97%).

The tizoxanide and pyridine are linked through hydrogen bond O1–H···N8 (bond distance = 2.567 Å). A stable crystal was formed through intramolecular O1—H···N7 (bond distance = 2.625 Å) and intermolecular O1—H···N8 hydrogen-bonding interactions involving the benzamide group and the pyridine molecule. It is arresting that π–π interactions play an important role in the molecular packing. Inversion-related pyridine molecules are linked by π-π interactions [centroid-centroid distance = 3.803 (6) Å], which stabilize the crystal. By comparison with X-ray of prodrug nitazoxanide (Bruno *et al.*, 2010), tizoxanide has stronger intramolecular hydrogen bonds. These hydrogen bonds may be useful for pharmaceutical preparation of tizoxanide. The molecular and crystal structures are stabilized by intra- (O1—H···N7) and intermolecular (O1—H···N8, π-stacking) interactions respectively, which give a great stability to the crystal building.

When the drug crystallized from different solvents, the crystal form may be changed and then altering a drug's properties, such as melting point and solubility (Trask *et al.*, 2004). We speculated that the replacement of a weak alkaline solvent or solid compound containing the pyridine ring may form the corresponding crystals made of different formulations of active drugs for tizoxanide. Obviously, crystal form of the title compound is different from prodrug nitazoxanide, which suggests that changing solvent or pyridine derivatives may form new crystals and new dosage forms of drugs. This is our future work.

### Experimental

A solution of 80 mg of nitazoxanide in 250 ul of pyridine was stirred for 15 min at 333 K and left to crystallize at 293 K overnight. A size suitable flaxen needle was attained for X-ray analysis.

Thermogravimetric analysis for the title complex was performed using a NETZSCH STA409 instrument with sample. The sample was placed in an aluminium cell, heated at 5 °C min^-1^ and purged with nitrogen gas flowing at 20 cm^3^ min^-1^.

### Crystal data

**Table:**

C_10_H_7_N_3_O_4_S·C_5_H_5_N	*Z* = 2
*M**_r_* = 344.35	*F*(000) = 356
Triclinic, *P*1	*D*_x_ = 1.497 Mg m^−^^3^
*a* = 6.9826 (3) Å	Cu *K*α radiation, λ = 1.5418 Å
*b* = 10.0462 (5) Å	Cell parameters from 3840 reflections
*c* = 11.8387 (7) Å	θ = 3.9–65.7°
α = 102.998 (5)°	µ = 2.16 mm^−^^1^
β = 99.037 (5)°	*T* = 293 K
γ = 104.367 (4)°	Rod, colorless
*V* = 763.69 (7) Å^3^	0.20 × 0.15 × 0.10 mm

### Data collection

**Table:**

Agilent Xcalibur Onyx Nova diffractometer	2481 independent reflections
Radiation source: Nova (Cu) X-ray Source	2349 reflections with *I* > 2σ(*I*)
Mirror monochromator	*R*_int_ = 0.016
Detector resolution: 8.2417 pixels mm^-1^	θ_max_ = 64.0°, θ_min_ = 3.9°
ω scans	*h* = −4→8
Absorption correction: multi-scan (*CrysAlis PRO*; Agilent, 2011)	*k* = −11→11
*T*_min_ = 0.712, *T*_max_ = 0.806	*l* = −13→13
4493 measured reflections	

### Refinement

**Table:**

Refinement on *F*^2^	Primary atom site location: structure-invariant direct methods
Least-squares matrix: full	Secondary atom site location: difference Fourier map
*R*[*F*^2^ > 2σ(*F*^2^)] = 0.028	Hydrogen site location: inferred from neighbouring sites
*wR*(*F*^2^) = 0.075	All H-atom parameters refined
*S* = 1.08	*w* = 1/[σ^2^(*F*_o_^2^) + (0.0385*P*)^2^ + 0.2467*P*] where *P* = (*F*_o_^2^ + 2*F*_c_^2^)/3
2481 reflections	(Δ/σ)_max_ = 0.001
265 parameters	Δρ_max_ = 0.20 e Å^−^^3^
0 restraints	Δρ_min_ = −0.25 e Å^−^^3^

### Special details

**Table:**

Experimental. Empirical absorption correction using spherical harmonics, implemented in SCALE3 ABSPACK scaling algorithm.
Geometry. All e.s.d.'s (except the e.s.d. in the dihedral angle between two l.s. planes) are estimated using the full covariance matrix. The cell e.s.d.'s are taken into account individually in the estimation of e.s.d.'s in distances, angles and torsion angles; correlations between e.s.d.'s in cell parameters are only used when they are defined by crystal symmetry. An approximate (isotropic) treatment of cell e.s.d.'s is used for estimating e.s.d.'s involving l.s. planes.
Refinement. Refinement of *F*^2^ against ALL reflections. The weighted *R*-factor *wR* and goodness of fit *S* are based on *F*^2^, conventional *R*-factors *R* are based on *F*, with *F* set to zero for negative *F*^2^. The threshold expression of *F*^2^ > σ(*F*^2^) is used only for calculating *R*-factors(gt) *etc*. and is not relevant to the choice of reflections for refinement. *R*-factors based on *F*^2^ are statistically about twice as large as those based on *F*, and *R*- factors based on ALL data will be even larger.

### Fractional atomic coordinates and isotropic or equivalent isotropic displacement parameters (Å^2^)

**Table:**

	*x*	*y*	*z*	*U*_iso_*/*U*_eq_	
S1	0.13956 (5)	0.44049 (4)	−0.18461 (3)	0.02193 (13)	
O2	0.38793 (16)	0.69194 (12)	−0.07942 (9)	0.0287 (3)	
O4	−0.09371 (17)	0.24233 (13)	−0.40776 (9)	0.0379 (3)	
O5	−0.23353 (17)	0.06550 (12)	−0.34113 (10)	0.0375 (3)	
N6	−0.12170 (18)	0.18630 (14)	−0.32633 (11)	0.0274 (3)	
N7	0.31757 (17)	0.56377 (13)	0.04990 (11)	0.0200 (3)	
N8	0.59845 (19)	0.69146 (13)	0.49121 (11)	0.0275 (3)	
C9	0.5749 (2)	0.80811 (15)	0.24522 (13)	0.0208 (3)	
C10	0.5468 (2)	0.80762 (15)	0.12457 (12)	0.0201 (3)	
N11	0.09847 (18)	0.33048 (13)	−0.00698 (11)	0.0225 (3)	
C12	0.4136 (2)	0.68695 (15)	0.02405 (12)	0.0208 (3)	
C13	−0.0197 (2)	0.22865 (17)	−0.10729 (14)	0.0241 (3)	
C14	−0.0158 (2)	0.26888 (16)	−0.20893 (13)	0.0230 (3)	
C15	0.8152 (2)	1.04417 (17)	0.29859 (14)	0.0290 (4)	
C16	0.1899 (2)	0.44557 (15)	−0.03509 (12)	0.0196 (3)	
C17	0.7287 (2)	0.61931 (18)	0.51622 (16)	0.0344 (4)	
C18	0.5727 (3)	0.7465 (2)	0.69334 (16)	0.0470 (5)	
C19	0.6541 (2)	0.92756 (16)	0.09458 (14)	0.0252 (3)	
C20	0.7871 (2)	1.04502 (17)	0.17969 (15)	0.0296 (4)	
C21	0.5227 (3)	0.75377 (18)	0.57844 (15)	0.0354 (4)	
C22	0.7067 (3)	0.6728 (2)	0.71945 (17)	0.0518 (6)	
C23	0.7866 (3)	0.6082 (2)	0.6301 (2)	0.0489 (5)	
C24	0.7100 (2)	0.92842 (16)	0.33129 (14)	0.0251 (3)	
O1	0.47188 (15)	0.69476 (11)	0.27650 (9)	0.0237 (2)	
H19	0.631 (3)	0.9235 (18)	0.0134 (17)	0.029 (4)*	
H24	0.725 (3)	0.9313 (19)	0.4158 (17)	0.032 (4)*	
H20	0.857 (3)	1.127 (2)	0.1564 (17)	0.040 (5)*	
H13	−0.093 (3)	0.138 (2)	−0.1026 (15)	0.028 (4)*	
H15	0.906 (3)	1.122 (2)	0.3591 (17)	0.035 (5)*	
H7	0.332 (3)	0.561 (2)	0.1221 (18)	0.033 (5)*	
H17	0.779 (3)	0.575 (2)	0.4501 (18)	0.040 (5)*	
H21	0.427 (3)	0.805 (2)	0.5559 (19)	0.052 (6)*	
H22	0.739 (4)	0.670 (3)	0.799 (2)	0.075 (7)*	
H18	0.514 (4)	0.800 (3)	0.762 (2)	0.068 (7)*	
H23	0.877 (3)	0.558 (2)	0.641 (2)	0.056 (6)*	
H25	0.525 (4)	0.698 (3)	0.355 (2)	0.073 (8)*	

### Atomic displacement parameters (Å^2^)

**Table:**

	*U*^11^	*U*^22^	*U*^33^	*U*^12^	*U*^13^	*U*^23^
S1	0.0230 (2)	0.0258 (2)	0.01336 (19)	0.00558 (15)	0.00094 (14)	0.00226 (14)
O2	0.0343 (6)	0.0325 (6)	0.0151 (5)	0.0044 (5)	0.0018 (4)	0.0073 (4)
O4	0.0353 (6)	0.0502 (7)	0.0181 (6)	0.0042 (5)	0.0012 (5)	0.0023 (5)
O5	0.0310 (6)	0.0309 (6)	0.0347 (7)	−0.0005 (5)	−0.0007 (5)	−0.0057 (5)
N6	0.0209 (6)	0.0329 (8)	0.0206 (7)	0.0062 (6)	0.0005 (5)	−0.0030 (6)
N7	0.0223 (6)	0.0237 (6)	0.0121 (6)	0.0062 (5)	0.0014 (5)	0.0033 (5)
N8	0.0310 (7)	0.0258 (7)	0.0186 (6)	−0.0012 (5)	0.0002 (5)	0.0063 (5)
C9	0.0200 (7)	0.0237 (7)	0.0195 (7)	0.0088 (6)	0.0045 (6)	0.0049 (6)
C10	0.0200 (7)	0.0232 (7)	0.0176 (7)	0.0096 (6)	0.0037 (6)	0.0035 (6)
N11	0.0227 (6)	0.0238 (6)	0.0200 (6)	0.0071 (5)	0.0034 (5)	0.0044 (5)
C12	0.0194 (7)	0.0249 (8)	0.0186 (7)	0.0086 (6)	0.0038 (6)	0.0051 (6)
C13	0.0208 (7)	0.0237 (8)	0.0249 (8)	0.0062 (6)	0.0032 (6)	0.0027 (6)
C14	0.0192 (7)	0.0255 (8)	0.0197 (7)	0.0064 (6)	0.0010 (6)	−0.0006 (6)
C15	0.0291 (8)	0.0231 (8)	0.0263 (8)	0.0031 (7)	0.0008 (7)	−0.0009 (7)
C16	0.0186 (7)	0.0247 (7)	0.0158 (7)	0.0097 (6)	0.0031 (5)	0.0030 (6)
C17	0.0292 (8)	0.0318 (9)	0.0379 (10)	0.0005 (7)	0.0043 (7)	0.0128 (8)
C18	0.0652 (13)	0.0402 (10)	0.0225 (9)	−0.0032 (9)	0.0079 (9)	0.0052 (8)
C19	0.0273 (8)	0.0267 (8)	0.0220 (8)	0.0086 (6)	0.0044 (6)	0.0078 (6)
C20	0.0320 (8)	0.0247 (8)	0.0300 (9)	0.0047 (7)	0.0058 (7)	0.0084 (7)
C21	0.0433 (10)	0.0320 (9)	0.0248 (9)	0.0046 (8)	0.0050 (7)	0.0047 (7)
C22	0.0655 (13)	0.0449 (11)	0.0231 (10)	−0.0172 (10)	−0.0096 (9)	0.0168 (9)
C23	0.0374 (10)	0.0422 (11)	0.0595 (14)	−0.0019 (9)	−0.0110 (9)	0.0292 (10)
C24	0.0268 (8)	0.0264 (8)	0.0187 (8)	0.0074 (6)	0.0022 (6)	0.0018 (6)
O1	0.0269 (5)	0.0253 (6)	0.0146 (5)	0.0025 (4)	0.0018 (4)	0.0048 (4)

### Geometric parameters (Å, º)

**Table:**

S1—C14	1.7269 (15)	C13—H13	0.944 (18)
S1—C16	1.7363 (14)	C15—C20	1.393 (2)
O2—C12	1.2240 (18)	C15—C24	1.382 (2)
O4—N6	1.2381 (17)	C15—H15	0.94 (2)
O5—N6	1.2265 (18)	C17—C23	1.383 (3)
N6—C14	1.4217 (19)	C17—H17	0.97 (2)
N7—C12	1.3775 (19)	C18—C21	1.373 (3)
N7—C16	1.3684 (19)	C18—C22	1.369 (3)
N7—H7	0.85 (2)	C18—H18	1.06 (2)
N8—C17	1.334 (2)	C19—C20	1.377 (2)
N8—C21	1.335 (2)	C19—H19	0.939 (18)
C9—C10	1.410 (2)	C20—H20	0.97 (2)
C9—C24	1.403 (2)	C21—H21	0.98 (2)
C9—O1	1.3483 (18)	C22—C23	1.381 (3)
C10—C12	1.481 (2)	C22—H22	0.94 (3)
C10—C19	1.403 (2)	C23—H23	0.92 (2)
N11—C13	1.365 (2)	C24—H24	0.983 (19)
N11—C16	1.3148 (19)	O1—H25	0.93 (3)
C13—C14	1.355 (2)		
			
C14—S1—C16	86.32 (7)	N11—C16—S1	116.83 (11)
O4—N6—C14	116.98 (13)	N11—C16—N7	121.36 (13)
O5—N6—O4	124.16 (13)	N8—C17—C23	121.43 (18)
O5—N6—C14	118.86 (13)	N8—C17—H17	116.3 (11)
C12—N7—H7	119.5 (13)	C23—C17—H17	122.3 (11)
C16—N7—C12	123.00 (12)	C21—C18—H18	121.1 (13)
C16—N7—H7	117.4 (13)	C22—C18—C21	118.52 (19)
C17—N8—C21	118.94 (14)	C22—C18—H18	120.4 (13)
C24—C9—C10	118.84 (13)	C10—C19—H19	116.6 (11)
O1—C9—C10	120.13 (13)	C20—C19—C10	121.77 (14)
O1—C9—C24	121.02 (13)	C20—C19—H19	121.6 (11)
C9—C10—C12	124.80 (13)	C15—C20—H20	121.2 (11)
C19—C10—C9	118.97 (13)	C19—C20—C15	118.89 (15)
C19—C10—C12	116.22 (13)	C19—C20—H20	119.8 (11)
C16—N11—C13	109.77 (12)	N8—C21—C18	122.73 (18)
O2—C12—N7	119.41 (13)	N8—C21—H21	116.0 (13)
O2—C12—C10	123.02 (13)	C18—C21—H21	121.2 (13)
N7—C12—C10	117.56 (12)	C18—C22—C23	119.32 (17)
N11—C13—H13	120.4 (11)	C18—C22—H22	116.7 (16)
C14—C13—N11	114.35 (14)	C23—C22—H22	124.0 (16)
C14—C13—H13	125.2 (11)	C17—C23—H23	116.4 (15)
N6—C14—S1	120.07 (11)	C22—C23—C17	119.06 (19)
C13—C14—S1	112.72 (11)	C22—C23—H23	124.5 (15)
C13—C14—N6	127.21 (14)	C9—C24—H24	119.1 (11)
C20—C15—H15	121.3 (11)	C15—C24—C9	120.72 (14)
C24—C15—C20	120.79 (15)	C15—C24—H24	120.1 (11)
C24—C15—H15	117.9 (11)	C9—O1—H25	111.8 (16)
N7—C16—S1	121.81 (11)		

### Hydrogen-bond geometry (Å, º)

**Table:**

*D*—H···*A*	*D*—H	H···*A*	*D*···*A*	*D*—H···*A*
N7—H7···O1	0.85 (2)	1.95 (2)	2.6248 (16)	135.9 (18)
O1—H25···N8	0.94 (2)	1.64 (2)	2.5671 (16)	175 (3)
